# Integrative analysis of DNA methylation and gene expression reveals key molecular signatures in acute myocardial infarction

**DOI:** 10.1186/s13148-022-01267-x

**Published:** 2022-03-27

**Authors:** Xiaoli Luo, Yi Hu, Junwei Shen, Xinwen Liu, Tao Wang, Li Li, Jue Li

**Affiliations:** 1 Key Laboratory of Arrhythmias of the Ministry of Education of China, East Hospital, Tongji University School of Medicine, Shanghai, 200120 China; 2 School of Medicine, Tongji University, No. 1239, Siping Road, Shanghai, 200092 China

**Keywords:** DNA methylation, MeDIP-seq, RNA-seq, Acute myocardial infarction, Gene regulation

## Abstract

**Backgrounds:**

Acute myocardial infarction (AMI) has been one of the most fatal diseases among all types of heart diseases due to its rapid onset and high rates of fatality. Understanding accurately how multi-omics molecular features change at the early stage of AMI is crucial for its treatment. Currently, the changes involved in DNA methylation modification and gene expression of multiple genes have remained unexplored.

**Results:**

We used the RNA-seq and MeDIP-seq on heart tissues from AMI mouse models at series of time points (Sham, AMI 10-min, 1-h, 6-h, 24-h and 72-h), to comprehensively describe the transcriptome and genome-wide DNA methylation changes at above time points. We identified 18814, 18614, 23587, 26018 and 33788 differential methylation positions (DMPs) and 123, 135, 731, 1419 and 2779 differentially expressed genes (DEGs) at 10-min, 1-h, 6-h, 24-h and 72-h AMI, respectively, compared with the sham group. Remarkably, the 6-h AMI with the drastic changes of DEGs and a large number of enriched functional pathways in KEGG may be the most critical stage of AMI process. The 4, 9, 40, 26, and 183 genes were further identified at each time point, based on the negative correlation (*P* < 0.05) between the differential mRNA expression and the differential DNA methylation. The mRNA and the promoter methylation expressions of five genes (*Ptpn6*, *Csf1r*, *Col6a1*, *Cyba*, and *Map3k14*) were validated by qRT-PCR and BSP methods, and the mRNA expressions were further confirmed to be regulated by DNA methylation in cardiomyocytes in vitro.

**Conclusions:**

Our findings profiled the molecular variations from the perspective of DNA methylation in the early stage of AMI and provided promising epigenetic-based biomarkers for the early clinical diagnosis and therapeutic targets of AMI.

**Supplementary Information:**

The online version contains supplementary material available at 10.1186/s13148-022-01267-x.

## Introduction

Acute myocardial infarction (AMI) remains one of the major causes of mortality and disability worldwide, resulting in considerable medical expenditure. AMI is manifested by the rupture of fragile plaques into the coronary artery, followed by coagulation and blockage of blood vessels, and ultimately leading to the death of massive myocardial cells [1]. The pathological process after AMI includes the early acute inflammation period and the late repair and remodeling period [2]. Understanding the molecular changes in the early stage of AMI is conducive to disease diagnosis and intervention.

A number of omics studies focused on the molecular mechanisms in transcription level involved in AMI. The changes of transcriptome in myocardial tissue despite depriving from infarct or survival area have been investigated via animal models of various periods after AMI [3–5]. Several studies also have identified highly potential molecular biomarkers for early diagnosis of AMI through bioinformatics analysis of RNA samples from the whole blood of patients [6, 7]. However, the molecular information available in existing studies is still limited for clinically effective and timely intervention of AMI. Epigenetics data, especially DNA methylation, are a promising way to understand molecular mechanisms of diseases. A few studies have paid attention to the connection between DNA methylation and AMI [8–10].

DNA methylation data usually evaluate the frequency of cytosine with methyl modification at the cytosine-phosphate-guanine (CpG) site, which plays an important role in the regulation of gene expression. Recent advances in scientific technology have allowed investigations to detect genome-wide DNA methylation status. It was revealed that differential DNA methylation at many CpG sites in blood sample was associated with a history of MI [11]. A prospective study indicated that blood-derived DNA methylation was associated with the risk of MI [12]. Ward-Cavines et al. have brought to light changes of DNA methylation after an incident MI in blood leukocytes from patients [13].

However, little is known about the alterations and functions of DNA methylation in myocardial tissues in the early stage of AMI, to date. To fill this gap, we conducted an integrative analysis of transcriptome and DNA methylome from heart tissues of AMI mouse models (0–72 h), to identify DNA methylation variations and their effects on gene expressions in the development of AMI and provide potential diagnostic markers and therapeutic targets from the perspective of DNA methylation.

## Results

### The landscape of DNA methylation modification changes during the progression of AMI

**Fig. 1:**
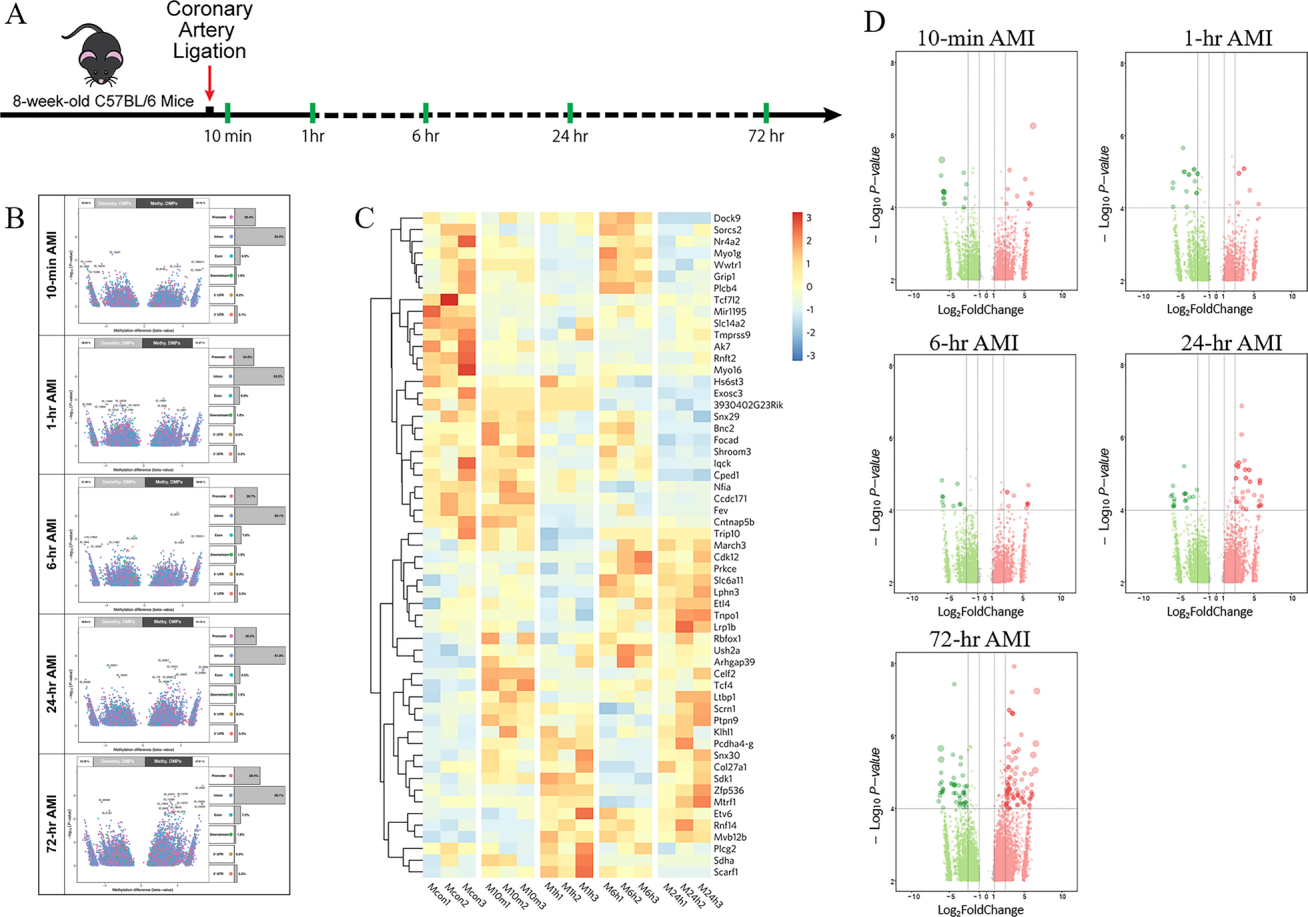
Genome-wide DNA methylation analysis of AMI models at series of time points. **A** Schematic diagram of the study design. **B** Volcano plot with the differential methylation sites (DMPs) (|log_2_FC|> 1; *P* value < 0.05). **C** Heatmap with the top 50 DMPs that appeared simultaneously at the five time points. **D** Volcano plot with DMPs in the promoter regions. FC, Fold Change; AMI, acute myocardial infarction

We generated the AMI mouse models by the ligation of the proximal left anterior descending coronary artery, and six time points were selected (Sham, AMI 10-min, 1-h, 6-h, 24-h and 72-h, n = 18) to evaluate the molecular changes in the process of AMI (Fig. 1A). At each time point, the infarcted left ventricular tissues from AMI mice were harvested for RNA-seq and MeDIP-seq. The effectiveness of the mouse models in simulating AMI process has been proved by cardiac ultrasound in the published work by our team [14].

To explore the relationship between DNA methylation modifications and AMI progress, we used the Peaks location information obtained from the MeDIP-seq sequencing data to analyze Peaks, genes and gene functional elements (Promoter, 5’ UTR, CDS, 3’ UTR, Intron and TTR). The CpG islands were annotated to detect the distribution of methylation-enriched regions on the genomic elements (Additional file 1: Figure S1). We found that the overall distribution of Peaks on the genome did not change much in different time points, but it should be some of the minor differences that lead to huge changes in the transcriptional level of genes. In order to obtain the methylation sites related to the development of AMI, we used the sham group as a control to perform differential DNA methylation analysis in different time points post-AMI (|log_2_FC|> 1; *P* value < 0.05). As shown in Fig. 1B and Additional file 2, we obtained 18814, 18614, 23587, 26018 and 33788 differential methylation positions (DMPs) at 10-min, 1-h, 6-h, 24-h and 72-h AMI, respectively. And there were 7951(42%), 9108(49%), 1212(51%), 12707 (49%), 13631 (52%) hypomethylation sites of DMPs, respectively (Fig. 1B). From the temporal distribution of DMPs, we found that the numbers and distinctions of DMPs were getting larger with the gradual progress of AMI. Figure 1C shows the top 50 DMPs that appeared simultaneously at these 5 time points.

To visualize the overall expression patterns of DMPs in different time points, we performed the hierarchical clustering analysis of all DMPs around different regions, respectively (Additional file 1: Figure S2). The Promoter and Exon regions showed similar clustering results that no significant difference existed in the methylation expression patterns between 10-min and sham groups, while distinct differences appeared at 1-h, 6-h, 24-h, and changed again at 72-h. The Intron and 3'UTR regions showed that 6-h was clustered into a separate category from 1 h and 24-h, suggesting these two methylation regions did not vary monotonically with time. In addition, the distal intergenic has more independent DNA methylation patterns among different time points, while the clustering effect of the 5'UTR region was not obvious.

A large number of studies have demonstrated that DNA methylation in the promoter region affects gene expression [15], so we specially selected the CpG islands in the promoter regions for further analysis, and identified 2638, 2477, 2979, 3704 and 5881 DMPs at the set time points post-AMI, respectively (Fig. 1D).

### Genes differentially expressed become evident in the early AMI phase

**Fig. 2:**
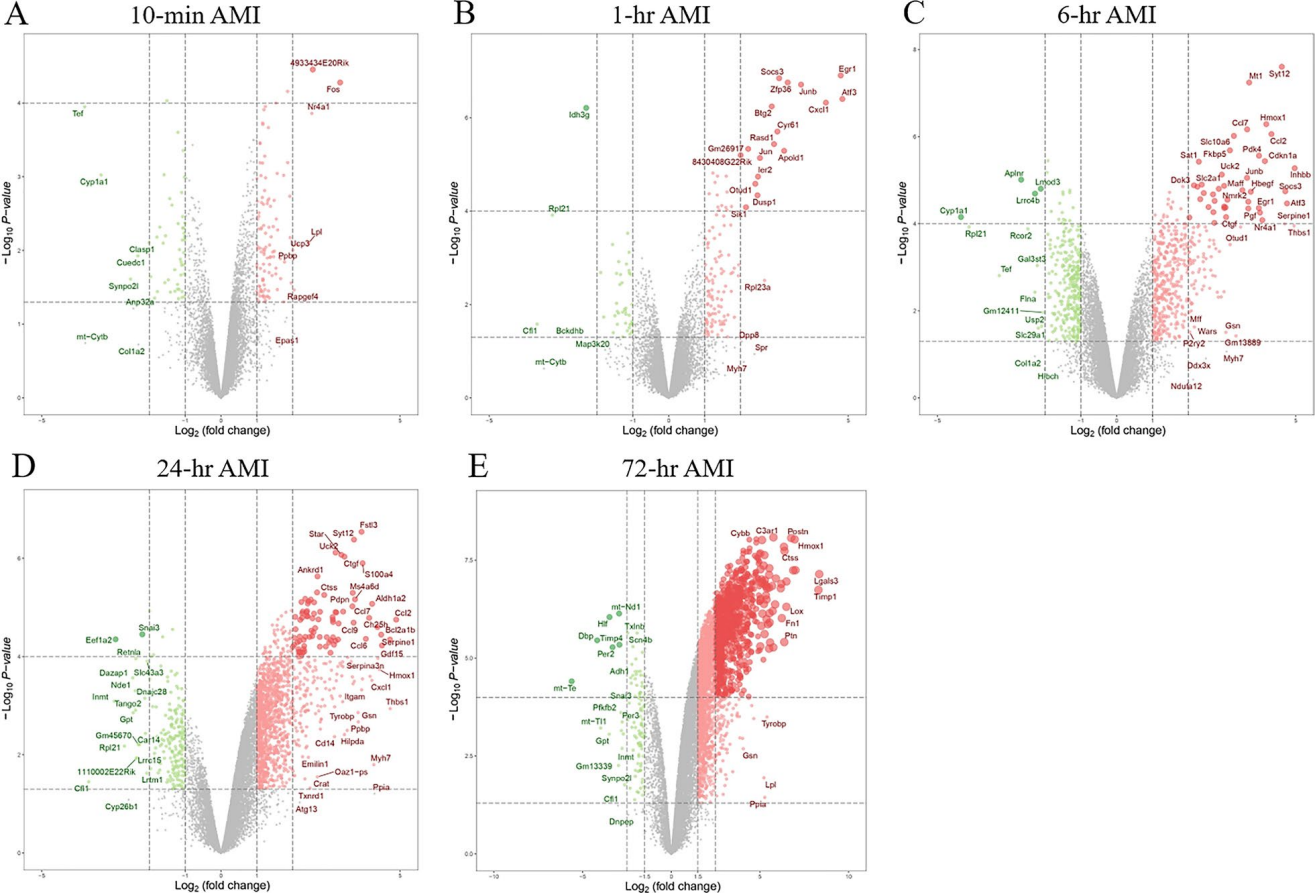
Differentially expressed genes (DEGs) analysis of transcriptome data. **A**–**E** Volcano plot with the DEGs (|log_2_FC|> 1 and *P* value < 0.05 for 10-min 1-h, 6-h and 24-h; |log_2_FC|> 1.5 and *P* value < 0.05 for 72-h) in the different time points of AMI. FC, Fold Change; AMI, acute myocardial infarction

To determine the transcriptome alterations in the development of AMI, RNA-Seq was used to detect the mRNA expression in the same sample sets. Our published work has performed the principal component analysis (PCA) on the transcriptome profiles and indicated that the 6-h group, 24-h group and 72-h group are clearly separated from other groups, while the very early time points (10-min, 1-h) seem to be close to the sham group [14]. Here, we identified 123 differentially expressed genes (DEGs) in the 10-min AMI group compared with the control group (|log_2_FC|> 1 and *P* value < 0.05), of which 89 genes were upregulated, while the other 34 were downregulated (Fig. 2A). At the following four time points, there were 135, 731, 1419, and 2779 DEGs compared with the control group, respectively (|log_2_FC|> 1 and *P* value < 0.05 for 1-h, 6-h and 24-h; |log_2_FC|> 1.5 and *P* value < 0.05 for 72-h), and the expressions of most DEGs increased (Fig. 2B–E and Additional file 3). Besides, the results indicated that the number of DEGs and the degree of differences in transcriptional expression gradually increased over time after AMI. It is worth noting that the drastic changes of DEGs started to occur from 6-h post-AMI, while previous stages (10-min, 1-h) showed relatively milder changes.

### Dynamic changes of pathways at different time points in AMI

**Fig. 3:**
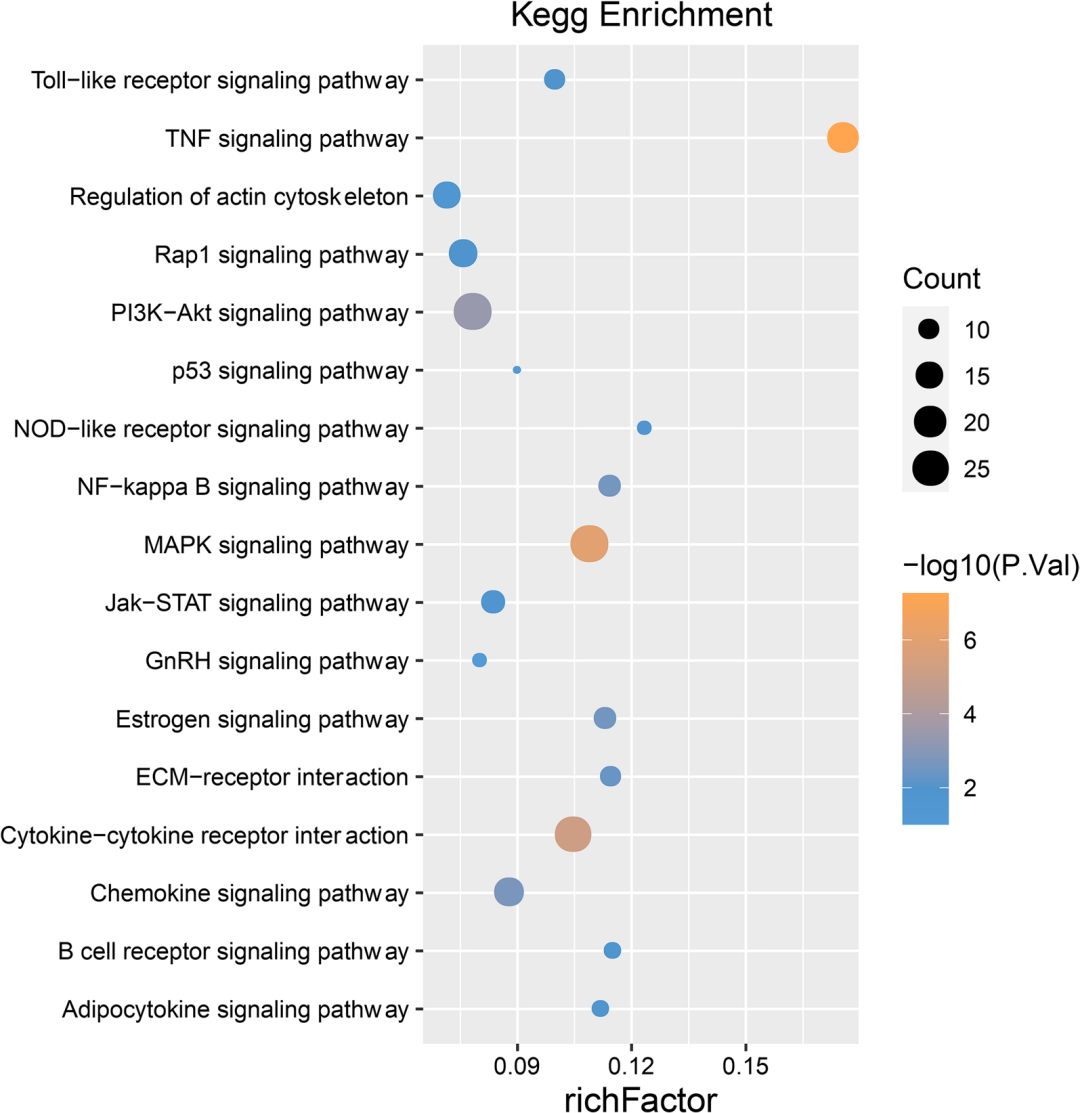
KEGG pathway enrichment analysis of differentially expressed genes at 6-h AMI

Next, the David gene function annotation online tool was used to perform pathway enrichment analysis on the screened DEGs at each time point. In the very early stage of AMI (10-min to 1-h), a small number of functional pathways were enriched (*P* < 0.05), including MAPK signaling pathway, TNF signaling pathway and PI3K-Akt signaling pathway, etc. (Additional file 1: Figure S3 A, B). At 6-h AMI, the structure of enriched pathways showed significant alterations that not only lots of new pathways such as Jak-STAT and Adipocytokine signaling pathway were added, but the range of DEGs involved in earlier enriched pathways expanded obviously (Fig. 3). It suggested that 6-h AMI was an important transformation period in the development of AMI. The most significant functional pathways contained TNF signaling pathway, MAPK signaling pathway and cytokine-cytokine receptor interaction, which were mainly associated with inflammation and immune and cellular process. From the stage of 24-h to 72-h AMI, metabolic pathways (Amino sugar and nucleotide sugar metabolism, Protein processing in endoplasmic reticulum) and disease pathways (Hypertrophic cardiomyopathy, Dilated cardiomyopathy) began to add to the enriched functional pathways (Additional file 1: Figure S3 C, D).

**Fig. 4:**
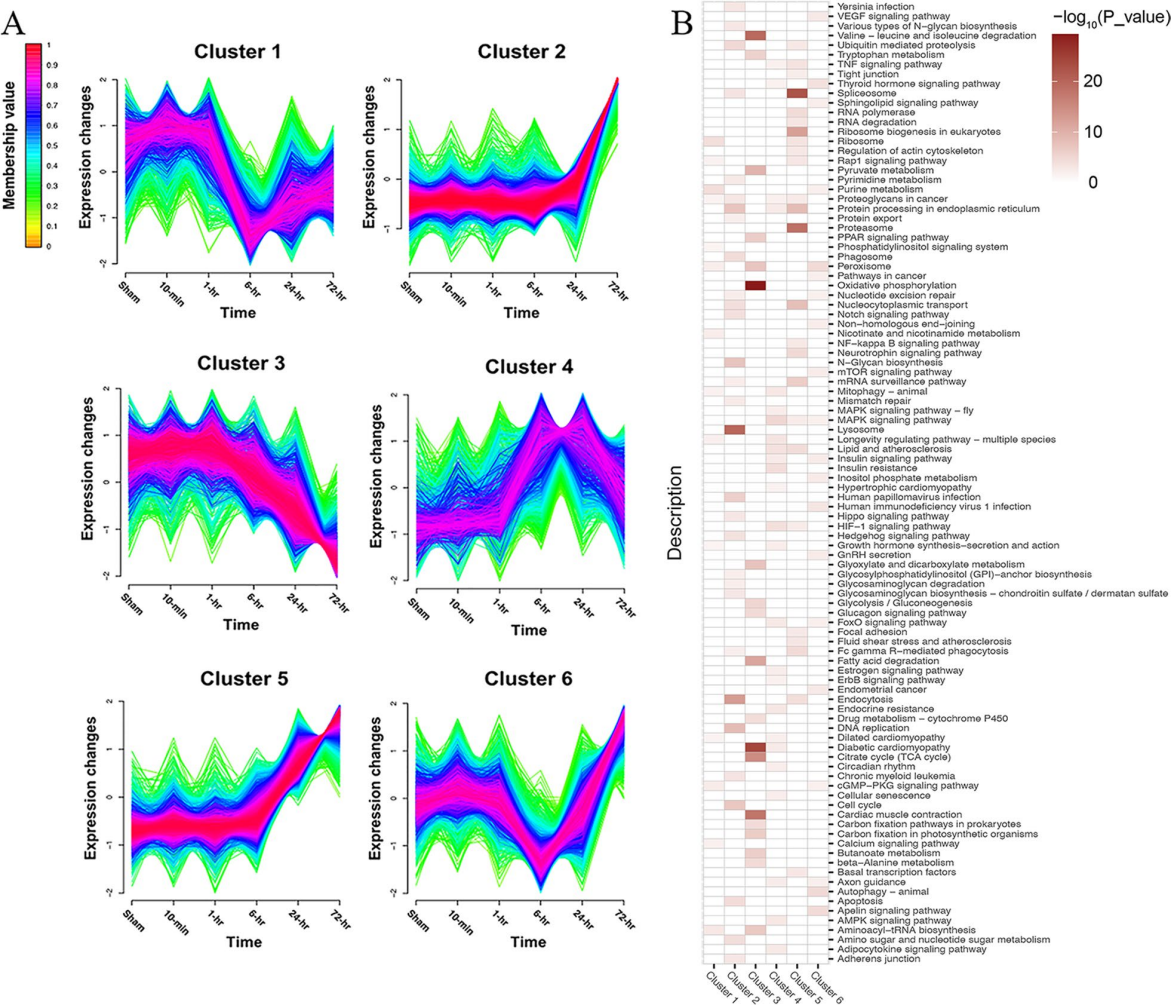
The clustering analysis of transcriptome dataset across 6 time points and the KEGG pathway enrichment analysis in each cluster

To define the temporal characteristics of the complete transcriptome dataset, we performed a clustering analysis of 10,765 genes across 6 time points using Mfuzz, which divided all the genes into 6 clusters (Fig. 4A). The genes in Cluster 2 and 5 both were monotonically upregulated over time, but Cluster 5 rose at 6-h earlier than Cluster 2 at 24-h, while the genes in Cluster 3 gradually downregulated overall. Then, we searched for enriched KEGG pathways (*P* value < 0.05) within each cluster and summarized these pathways via a heatmap (Fig. 4B). We found that the KEGG pathways were specific to one or more clusters. Some of the pathways enriched in Cluster 3 are unique such as "Oxidative phosphorylation" and "Cardiac muscle contraction," suggesting high temporal specificity with downtrend. On the contrary, there were more overlapping pathways in Cluster 2 and 5, such as "Spliceosome," "Endocytosis" and "Fc gamma R − mediated phagocytosis," indicating an upregulated trend over time.

### Identification of DEGs regulated by DMPs in the progression of AMI

**Fig. 5:**
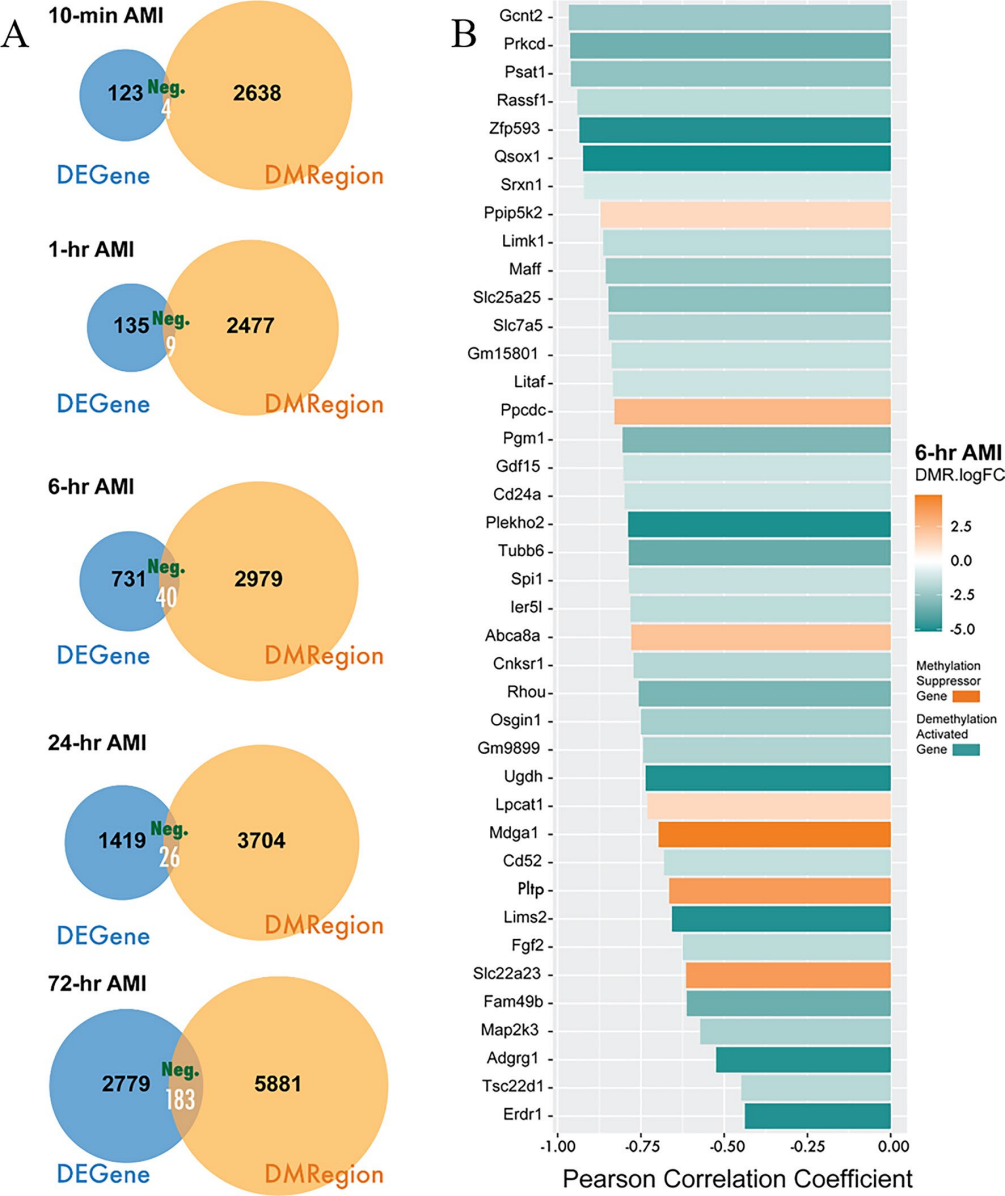
Correlation analysis of the DNA methylation level in promoter region and the mRNA expression. **A** The number of genes in the intersection of differentially expressed genes and differential DNA methylation in promoter region of genes, of which the DNA methylation and the mRNA expression were negatively correlated (Pearson Correlation Coefficient < 0 and *P* value < 0.05) for each time points. AMI, acute myocardial infarction; DEGene, differentially expressed gene; DMRegion, differentially methylation region; Neg, negative correlation.**B** 40 DEGs identified and their Pearson Correlation Coefficient at 6-h post-AMI

Hypermethylation at promoters tends to inhibit the expressions of downstream genes, while hypomethylation increases the expressions [15]. Here, we predicted DEGs regulated by DMPs in AMI process by the following criteria: (1) differential expression of mRNA, (2) differential methylation at promoters, (3) the levels of mRNA and methylation were negatively correlated via Pearson Correlation analysis (*P* value < 0.05 and Pearson Correlation Coefficient < 0). In the five time points (10-min, 1-h, 6-h, 24-h and 72-h) after AMI, 4, 9, 40, 26, and 183 genes were identified, respectively, that conform to the negative regulatory mechanism of DNA methylation (Fig. 5A and Additional file 4). Figure 5B shows the 40 DEGs regulated by DMPs at 6-h when the large-scale phenotypic changes brought about by DNA methylation started to happen after AMI. Furthermore, we found that most of these identified genes (33 of 40 genes) were highly expressed at transcription level with lower promoter methylation modifications, while the remaining small part of genes were downregulated with higher methylation levels.

### Function validation of DNA methylation modification on candidate genes

**Fig. 6:**
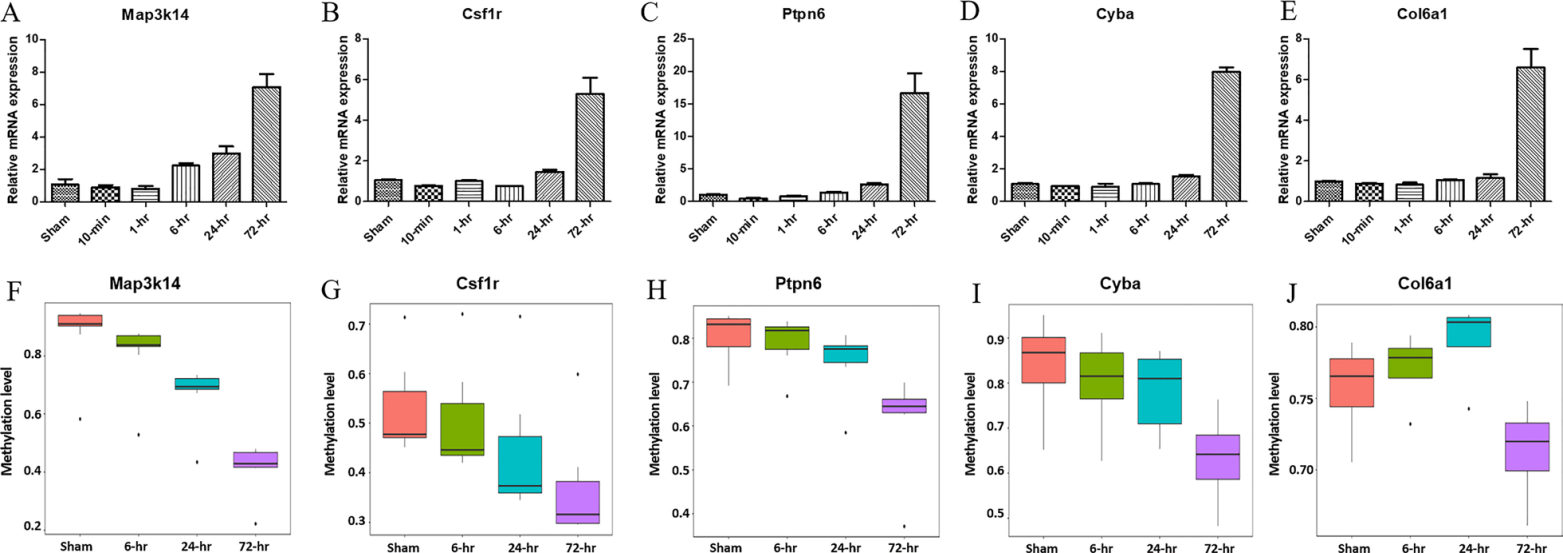
Expression validation of candidate genes. **A**–**E** Validation of the mRNA expression by qRT-PCR. **F**–**J** Validation of DNA methylation expression in the promoter regions by bisulfite sequencing PCR. The data are representative of three independent experiments

Based on above results, we performed expression and function validation of DNA methylation on candidate genes that might participate in essential biological process in AMI. First, we manually selected 32 genes which were predicted to be regulated by DNA methylation and involved in important KEGG pathways (Additional file 1: Table S1) to verify their mRNA expressions by qRT-PCR. As shown in Fig. 6A–E and Additional file 1: Figure S4, *Spi1, Map3k14, Ncf4*, etc., were upregulated at 6 h after MI, *Ptpn6, Plcg2, Edem1*, etc., were upregulated at 24 h, and *Cyba, Itgb5, Col6a1*, etc., were upregulated at 72 h.

Then, combining the results of qRT-PCR and MeDIP-Seq data, we excluded genes that the significant changes of DNA methylation occurred after changes in gene expression and then verified the DNA methylation status at promoters of the remaining 21 of above 32 genes using the next-generation sequencing-based BSP (Additional file 1: Table S2). The results showed that the trends in promoter methylation of 10 genes were consistent with predicted outcomes (Fig. 6F–J and Additional file 1: Figure S5). The methylation levels at promoter regions of *Ncf4, Map3k14,* and *Spi1* decreased at 6 h after MI, *Ptpn6, Csf1r* and *Itga11* decreased at 24 h, and *Ddost, Cyba, Itgb5, Col6a1* decreased at 72 h.

**Fig. 7:**
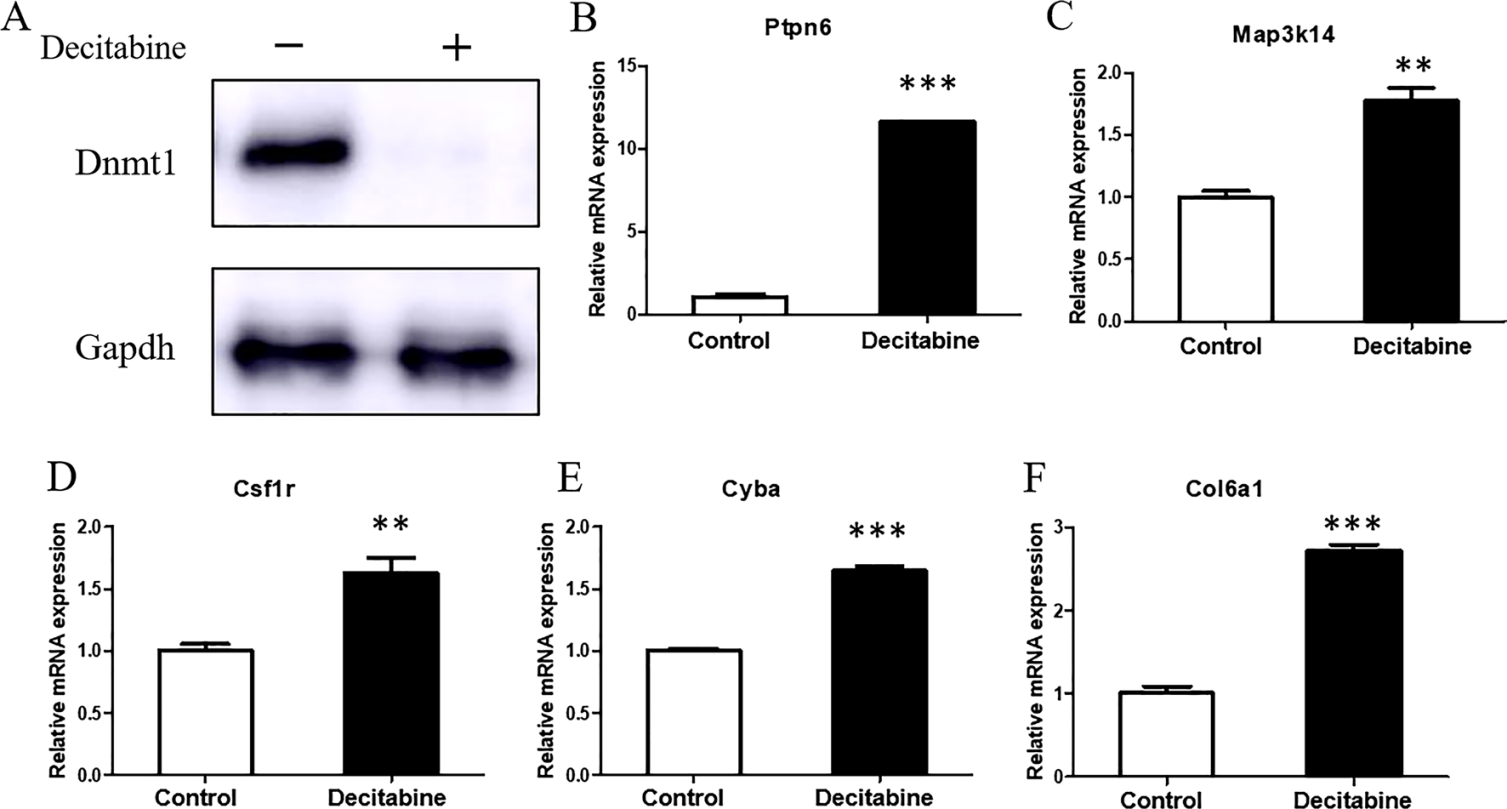
Validation of the genes regulated by DNA methylation in vitro. **A** The expression of methyltransferase 1 (*Dnmt1*) was inhibited by Decitabine in H9c2 cells. **B**–**F** The mRNA levels of candidate genes were upregulated after demethylation compared with the untreated group. The data are presented as the mean ± SD and representative of three independent experiments. **P < 0.01, ***P < 0.001

Furthermore, to address whether the transcription levels of these 10 genes were directly affected by DNA methylation in cardiomyocytes, the whole DNA methylation in H9c2 cells were inhibited in vitro. As shown in Fig. 7, 1 μM Decitabine inhibited the expression of methyltransferase 1 (*Dnmt1*) in H9c2 cells, and the mRNA levels of *Ptpn6, Csf1r, Col6a1, Cyba,* and *Map3k14* were upregulated after demethylation compared with the untreated group. It indicated that the expressions of these 5 candidate genes were regulated by DNA methylation in cardiomyocytes. *Ptpn6, Csf1r, Col6a1, Cyba,* and *Map3k14* have been involved in the pathogenesis of MI [16–20], so the methylation alterations at their promoters are supposed to play an essential role in AMI.

## Discussion

In summary, our study performed an integrative analysis of DNA methylation and mRNA expression datasets deriving from AMI mouse models at series of time points to identify key epigenetic alterations in AMI. It was found that 6-h AMI was the most critical stage in the development of AMI. Alterations at a large number of methylation modification sites occurred at this stage and affected the expression of downstream genes. In the Pearson correlation analysis, we have identified 33 highly expressed genes with hypomethylation as the demethylation activated genes and 7 low expressed genes with hypermethylation as the methylation suppressed genes. Based on the expression validation by qRT-PCR and bisulfite sequencing PCR, and the functional validation in vitro, we confirmed 5 candidate genes participating in the process of AMI through the regulation of DNA methylation. Our findings provided promising methylation-based biomarkers for the early clinical diagnosis and therapeutic targets of AMI.

The growing evidence has indicated that AMI was associated with epigenetic changes including DNA methylation. Abundant CpG sites were identified to be associated with the risk of incident coronary heart disease or MI [12]. And significant differences exist in DNA methylation profiles between pre- and post-MI [13]. Although methylation modifications have not yet been translated into clinical applications, researches on the biological function of specific methylation sites in MI have made remarkable progress, supporting the potentials as diagnostic markers and therapeutic targets. For example, regulation of methylation at *Runx3* affects cardiac microvascular endothelial cells injury and inflammatory response following MI [21]. Aspirin is widely used in the prevention of MI and one of the mechanisms is related to that methylation at *Fgf2* promoter modulated by aspirin protects human coronary artery endothelial cells against the effects of L5 [22].

Previous researches on the relationship between DNA methylation and MI usually used blood samples from patients as detection objects [11–13]. In view of the tissue-specific characteristics of epigenetic information, DNA methylation alterations in heart tissues should be more closely related to cardiac pathological mechanisms. A few specific DNA methylation sites have been previously explored via AMI animal models [23]. Here, our study systematically analyzed the genome-wide DNA methylation status, combined with transcriptome data, and finally identified a set of promoter sites at which methylation modifications changed and regulated the expression of downstream genes in AMI. Another strength of our study was to detect the methylation changes at series of time points in the early stage of AMI, which is difficult to sample in this way in population studies. The findings of animal studies could provide the basis for subsequent validation in human to generate clinical application value.

We validated the expressions of 32 genes involved in some enriched biological processes from the KEGG analysis, such as ECM-receptor interaction (*Itgb5, Col5a1, Col6a1*), phagosome (*Cyba, Tcirg1, Ncf4*) and osteoclast differentiation (*Csf1r, Plcg2, Spi1*). Many of these genes have been known to be associated with MI. It was reported that the protein kinase C-delta (*Prkcd*) was upregulated post-AMI and mediated the myocardial ischemia/reperfusion injury and remodeling [24, 25]. Neutrophil Cytosolic Factor 4 (*Ncf4*) plays an important role in the innate immune response and has been implicated as a diagnostic biomarker and possible regulatory targets in AMI [26]. Some of the DEGs are new identified potential factors contributed to the pathology of AMI. Activation of *Csf1r/Plcg2* signaling pathway attenuates inflammation and thus regulates the pathogenesis of hypoxic-ischemic encephalopathy [27]. It is possible that *Csf1r/Plcg2* exerted the similar effects in MI that is also an ischemic disease. *Tubb6* (tubulin, beta 6 class V) is a member of the beta tubulin superfamily, which is linked to skeletal muscle, osteoclast function and brain tumors in previous studies [28–30]. *Tubb6* significantly increased only 6 h after MI, but its role in the development of AMI needs to be investigated in further research.

Five genes including *Ptpn6, Csf1r, Col6a1, Cyba* and *Map3k14* were identified as methylation-dependent transcriptional activated genes. *Ptpn6*, a tyrosine phosphatase protein, is known to be a signaling molecule that regulates a variety of cellular processes including apoptosis and proliferation. Increasing evidence indicates that *Ptpn6* is upregulated in early AMI and suppressing its expression through microRNA has cardioprotective effects via anti-apoptosis during induced AMI [16, 31]. In our study, it has been observed that the transcription level of *Ptpn6* in heart tissues increased significantly at 24-h after AMI, which was probably regulated by low methylation status at the promoter site. So, methylation modification in *Ptpn6* promoter region could be a new avenue to hinder the injury of AMI in the early stage.

In addition, the receptor for macrophage colony-stimulating factor (*Csf1r*) participants in modulating cardiac function post-MI for its contribution to the resolution of inflammation via M2-polarized macrophages [17, 32]. *Col6a1* encodes a type VI collagen which increased significantly after MI [18]. Mutation of *Col6a1* plays a protective role by improving cardiac remodeling and function in the MI mouse model [18]. p22phox encoded by *Cyba* gene is the regulatory subunit of NADPH oxidase and the gene polymorphism is involved in coronary artery disease including premature MI [19, 33]. *Map3k14* is involved in one of NF-κB pathways. NF-κB activation is an important event in inflammatory response that is the main pathophysiological process in the early stage of MI. Taken together, the DNA methylation alterations of these genes post-MI could be of important biological meaning. How these epigenetic modifications work on the pathogenesis of MI deserves further study.

The cardiac function and pathological manifestations of the mouse AMI models at 0–72 h have been described in the published article by our team [14]. The ejection fraction values in AMI mice dropped to about 30% at 24 h and 72 h, and the pathological examination showed that increased immune cells continuously infiltrated into the infarcted region post-AMI. In addition, other studies have proved that 3–4 days after AMI in mice belonged to the acute sterile inflammation period [2]. Functional enrichment analysis in this work also indicated that a large number of DEGs were enriched in immune and inflammatory pathways from 6-h post-AMI. So, we suppose that inflammation could be the reason for the major changes in DNA methylation and gene expression.

This study has several limitations. First, the time points of our experimental design were not completely continuous. Thus, although we found that 6-h post-MI was an important stage, the obvious changes in pathological molecules might have occurred before 6-h. Second, the sample size (3 per group) used for sequencing was relatively small, which might affect the efficiency of statistical analysis. Third, the five important methylation sites we identified need further experiments to clarify their biological functions in AMI. And future researches should be designed to test these DNA methylation biomarkers in blood for early clinical diagnostics of AMI, especially cell-free DNA in blood which could be released by injured tissues.

## Conclusions

Our study profiled the alterations of DNA methylation in myocardial tissues in the early stage of AMI by genome-wide methylation sequencing and revealed its influence on gene expression through integrated analysis with transcriptome. These findings provided potential biomarkers for the early clinical diagnosis and therapeutic targets of AMI from the perspective of DNA methylation.

## Methods

### Animal model

Specific pathogen-free C57BL/6 mice (male, 8 weeks) were purchased from Shanghai Slake Experimental Animal Co., LTD (Shanghai, China). The mice were randomly divided into different experimental groups (Sham, AMI 10-min, 1-h, 6-h, 24-h and 72-h) and each group had three mice. Mice were anesthetized with sodium pentobarbital (50 mg/kg, intraperitoneal injection). Following thoracotomy, the left anterior descending coronary artery was ligated to induce acute myocardial infarction. After MI for specific time, the mice were fully anesthetized with 1–3% isoflurane and then were euthanized via cervical dislocation. The infarcted heart tissues mainly in the ventricular apical regions were harvested for MeDIP-seq and RNA-seq. All animal experiments were approved by the Institutional Animal Care and Use Committee of Tongji University (Permit Number: TJAA08720101).

### MeDIP-seq data generation

The MeDIP-seq was conducted by Shanghai Biotechnology Corporation, China. Briefly, the DNA was sheared with Covaris S2 system and the target DNA size peak was 250 bp. Then, the end repair and adenylation of 3’ end treatments were performed. After the adaptor ligation reaction, the beads were used for fragment selection. Afterward, methylated DNA immunoprecipitation was carried out, and the eluted DNA was amplified with PCR to form the final sequencing library. The PCR products were purified and the library quality was assessed on the Agilent Bioanalyzer 2100. At last, the paired-end sequencing was performed on an Illumina NovoSeq6000.

### MeDIP-seq data processing and analysis

The read quality of sequencing was verified using the FastQC program (http://www.bioinformatics.babraham.ac.uk/projects/fastqc/). We selected Bowtie2 [34] as the mapping tool, and the reads for each MeDIP sample were mapped to the mouse (mm10) genome with default parameter options. The mapped read files were then converted to sorted and indexed BAM files using SAMtools. We use MACS2 [35] to detect the peak enrichment region of the bam file obtained in the above steps and get the peak locations and distributions on the chromosome (Promoter, 5′ UTR, CDS, 3′ UTR, Intron and TTR). The promoter region is 2000 bp upstream of the transcription start site and TTR is 5000 bp downstream of the transcription termination site. In the CpG islands, the CpG shore is 2000 bp upstream and downstream of CpG island and the CpG shelf is 2000 bp ~ 4000 bp range upstream and downstream of CpG island. To define differentially methylation positions (DMPs), the MEDIPS [36] R package was used to calculate differential coverage between control and other AMI groups. The edgeR [37] *P* value was used to determine the difference between two groups for each genomic window in this R package, and windows with an edgeR *P* value less than an arbitrarily selected threshold were considered DMPs. The threshold parameter setting is |log_2_FoldChange(FC)|> 1 and *P* value < 0.05, and DMPs are annotated, including peak positions and relative information.

### RNA-seq data analysis and functional annotation

The details about the RNA-seq data generation can be found in the published work by our team and the raw data were deposited in the Gene Expression Omnibus (GEO) (accession number GSE153494) [14]. The expression level for each gene was estimated with transcripts per million (TPM). Differentially expressed genes (DEGs) were analyzed following the workflow with HISAT2, StringTie, and Ballgown [38]. The clean data were aligned against the mouse reference genome (mm10) with HISAT2 [39]. The SAM files were sorted and converted to BAM files with SAMtools. Transcripts of each sample were assembled with StringTie [40] based on Mus_musculus_Ensemble_90.gtf (http://ftp.ensembl.org/pub/release-90/gtf/mus_musculus/) and BAM file for each sample. After assemblies, new generated GTF (General Transfer Format) documents and Mus_musculus_Ensemble_90.gtf were merged with the StringTie “--merge” option, and transcript abundances of each sample were estimated with the StringTie “-e -B” option [40]. The stattest function from Ballgown (https://bioconductor.org/packages/release/bioc/html/ballgown.html, Version 2.22.0) was used to screen the differentially expressed genes between the control group and other AMI groups. Transcripts with variance across samples less than one were prioritized removed. Genes with the significance threshold of |log_2_FC|> 1 and *P* value < 0.05 were identified as DEGs for groups of 10-min, 1-h, 6-h and 24-h AMI. To screen out more significant genes from a large number of genes for 72-h AMI, the threshold of DEGs was set more strictly as |log_2_FC|> 1.5 and *P* value < 0.05. We performed KEGG (Kyoto Encyclopedia of Genes and Genomes) pathway enrichment analysis [41] to investigate gene functions and signal pathways that may be involved in the occurrence and progressing of AMI with DAVID 6.8 (https://david.ncifcrf.gov/) [42].

### Correlation analysis

In order to investigate the association between DEGs and DMPs, Pearson correlation analysis was used to calculate the correlation between promoter methylation modification and gene expression for the methylation suppressed genes and demethylation activated genes. Based on the mechanism that DNA methylation modified promoter region can significantly inhibit gene expression [43], we used negative regulation as the screening criteria, and genes with correlation coefficient < 0 and *P* value < 0.05 were identified as the candidate genes.

### Next-generation sequencing-based bisulfite sequencing PCR

Gene-specific DNA methylation was assessed by a next-generation sequencing-based bisulfite sequencing PCR (BSP), according to previous protocols [44]. In brief, genomic DNA was converted using the ZYMO EZ DNA Methylation-Gold Kit (Zymo Research, Irvine, CA, USA) and one twentieth of the elution products were used as template. PCR amplification was conducted using KAPA 2G Robust HotStart PCR Kit (Kapa Biosystems, Wilmington, MA, USA). BSP products of multiple genes from one sample were generated, pooled equally and subjected to adaptor ligation. Barcoded libraries from all samples were sequenced on Illumina platform. The bisulfite sequencing reads were cleaned and aligned to the target sequences using software Bsmap (v2.73) with the default parameters. Methylation levels were calculated as the ratio of read counts of ‘C’ in the total read counts of both ‘C’ and ‘T’ for each covered C site.

### Quantitative reverse transcription-polymerase chain reaction (qRT-PCR)

Total RNA was isolated using Total RNA Extraction Reagent (Vazyme, Nanjing, China). Reverse transcription was performed by HiScript III RT SuperMix (Vazyme, Nanjing, China) according to the manufacturer’s instructions. The resulting cDNAs were detected using SYBR Green qPCR Master Mix (Biomake, Shanghai, China) and the Roche LightCycler 96 sequence detection system (Roche, Basel, Switzerland). The fold changes were calculated by 2−ΔΔCT method.

### Cell culture and treatment

H9c2 cell line of rat cardiac origin was cultured in Dulbecco's modified Eagle's medium (DMEM) supplemented with 10% fetal bovine serum, 100U/ml penicillin and 100ug/ml streptomycin, in a 5% CO2 humidified incubator at 37℃. To inhibited the DNA methylation, the cells were treated with 1 μM Decitabine (Selleck, Shanghai, China) for 48-h.

### Western blots

The H9C2 cells were lysed using RIPA Lysis Buffer (Beyotime, Nantong, China). Protein concentrations were detected using BCA Protein Assay Kit (Beyotime, Nantong, China). Western blot was performed using primary antibodies targeting to DNA methyltransferase 1 (Dnmt1) (Abcam, Cambridge, UK) and GAPDH (Proteintech, Chicago, USA). Blots were incubated with the HRP-linked secondary antibody. Analyses were performed with the Amersham Imager 600 (General Electric Company, Boston, USA).

### Statistical analysis

Three independent replicates were performed in each group for all experiments and data were expressed as the mean ± Standard Deviation (SD). To evaluate the statistical differences between the sham group and AMI groups, Student’s t test or Mann–Whitney test was used to calculate the *P* values, and *P* value < 0.05 was considered statistically significant difference. Pearson correlation coefficient was presented as the association of two independent features. All the statistical analysis was implemented using the R (4.0.1) programming language or GraphPad Prim 5.0 software.

## Supplementary Information

**Additional file 1**. Supplementary Figures and Tables.

**Additional file 2**. Differential methylation positions.

**Additional file 3**. Differential expressed genes.

**Additional file 4**. Differential expressed genes regulated by DNA methylation.

## Data Availability

The MeDIP-seq raw data reported in this paper have been deposited into the NCBI SRA database (https://www.ncbi.nlm.nih.gov/sra) with accession number PRJNA772355. The RNA-seq datasets analyzed during the current study are available in the Gene Expression Omnibus (GEO) (https://www.ncbi.nlm.nih.gov/geo/query/acc.cgi?acc=GSE153494) [14].

## Abbreviations

AMI: Acute myocardial infarction
DMPs: Differentially methylation positions
DEGs: Differentially expressed genes
FC: Fold Change
KEGG: Kyoto Encyclopedia of Genes and Genomes
qRT-PCR: Quantitative reverse transcription-polymerase chain reaction
CpG: Cytosine-phosphate-guanine
