# Supplementary material for: Integrative analysis of DNA methylation and gene expression reveals key molecular signatures in acute myocardial infarction
Source: Clin Epigenetics. 2022 Mar 27;14:46. doi: 10.1186/s13148-022-01267-x (PMC8958792; doi:10.1186/s13148-022-01267-x)
Supplement: Supplementary file 1 — Additional file 1. Supplementary Figures and Tables. [file 13148_2022_1267_MOESM1_ESM.docx]

**Supplementary materials**

**Sup. Figure S1.** The distribution of methylation-enriched regions on the genomic elements.


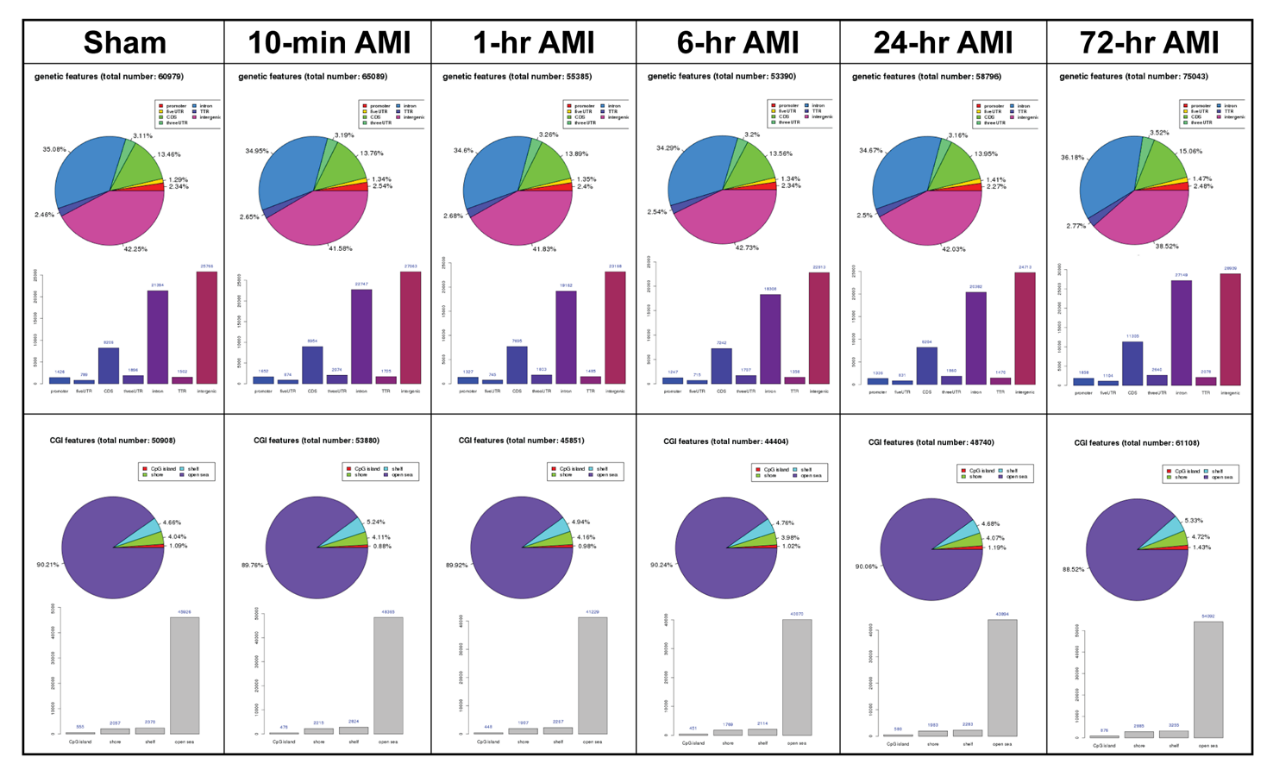


**Figure S2** Hierarchical clustering of DNA methylation at different time points around different sequence regions.


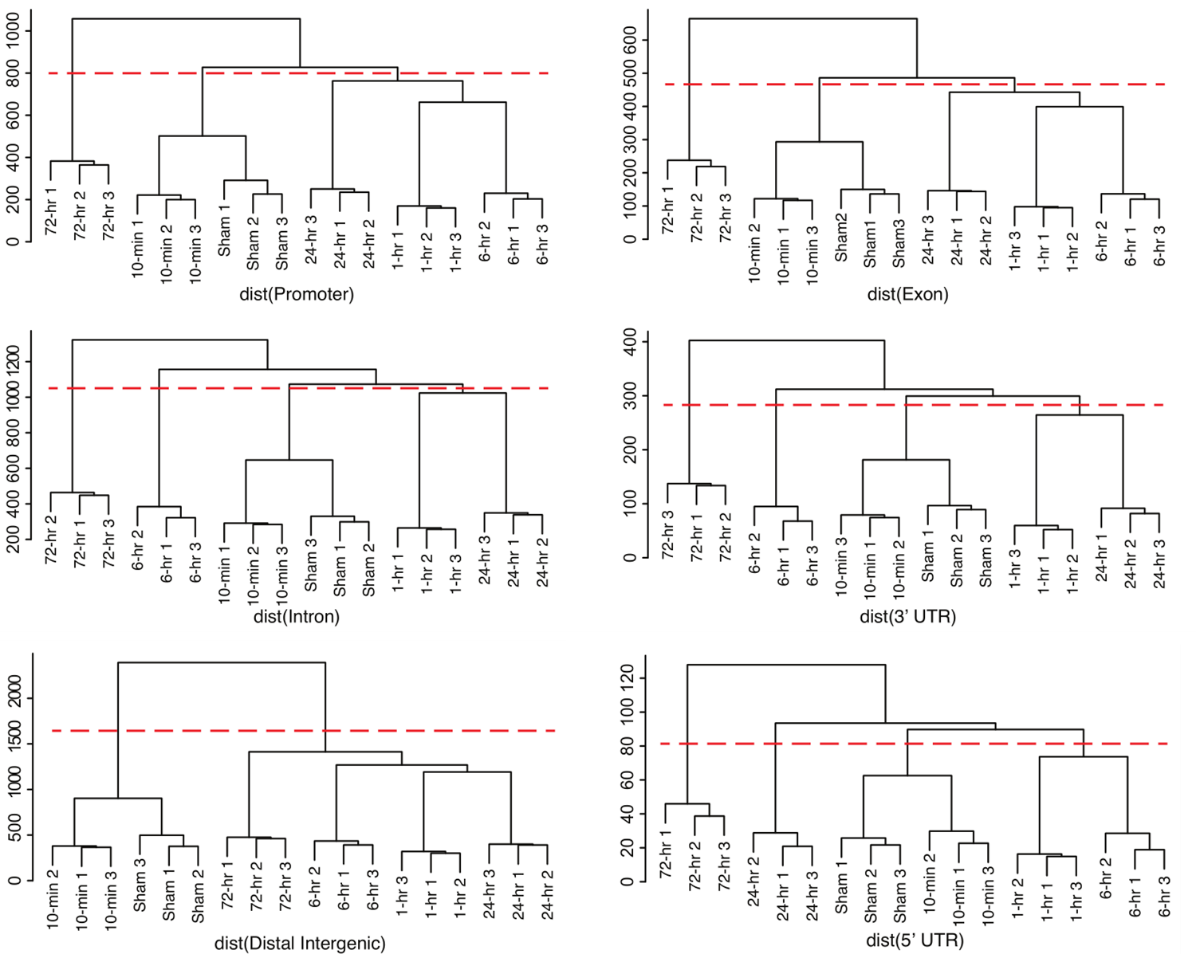


**Sup. Figure S3.** Pathway enrichment analysis of DEGs at different time points of AMI.


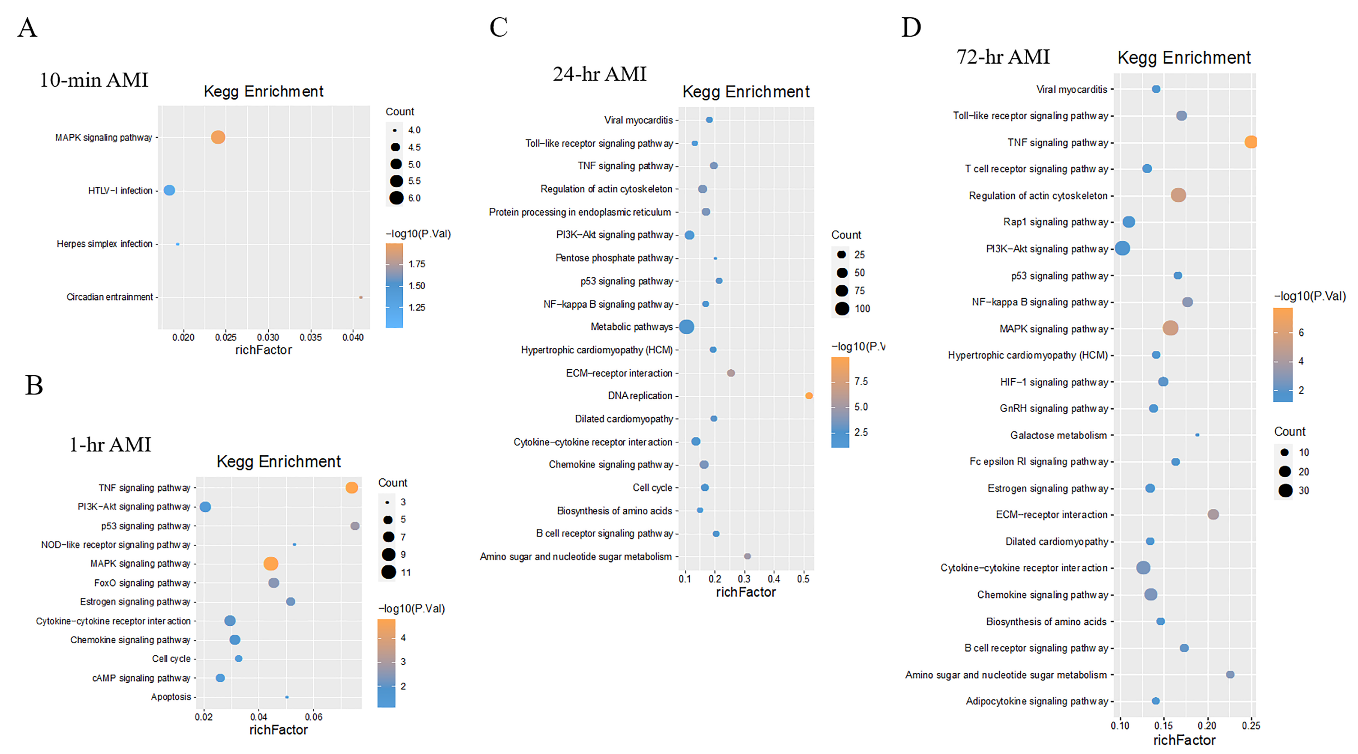


**Sup. Figure S4.** Validation of the mRNA expression of candidate genes by qRT-PCR.


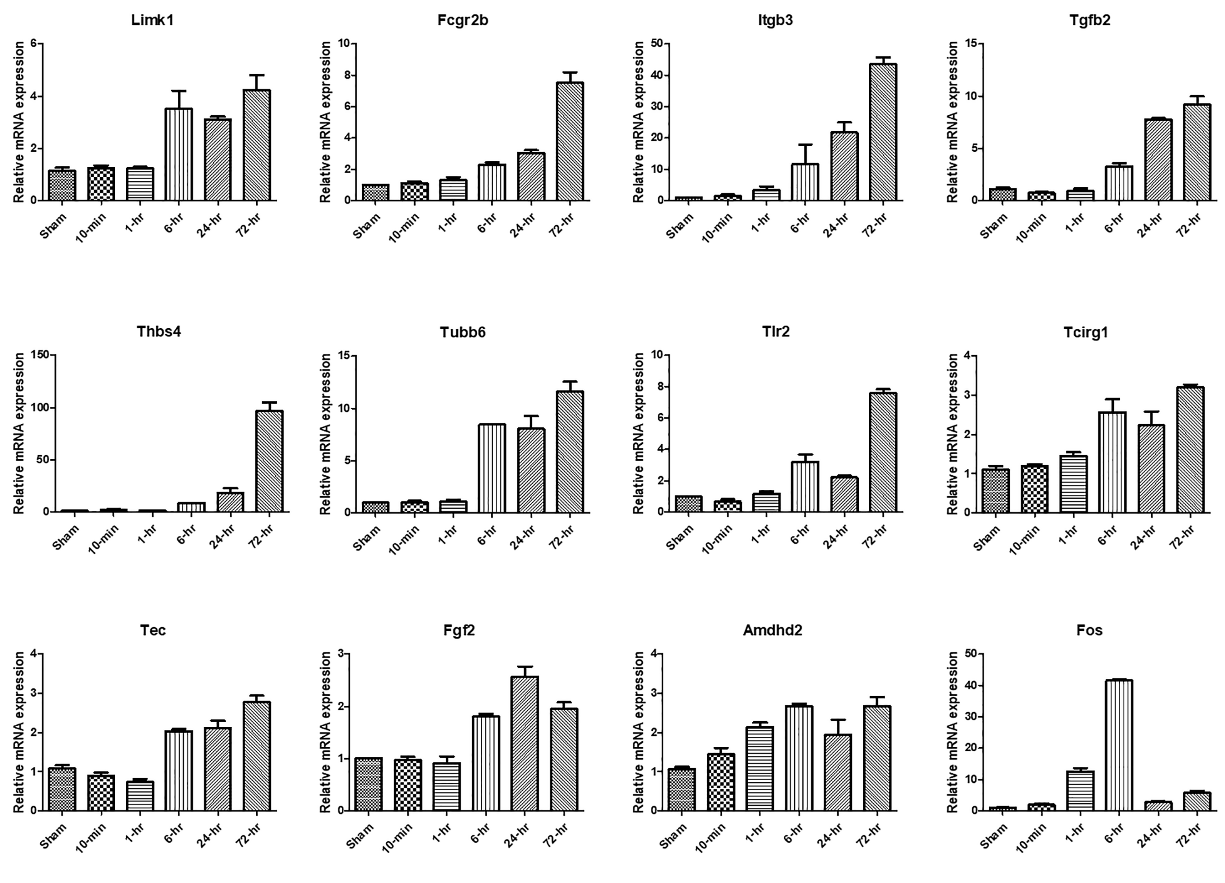


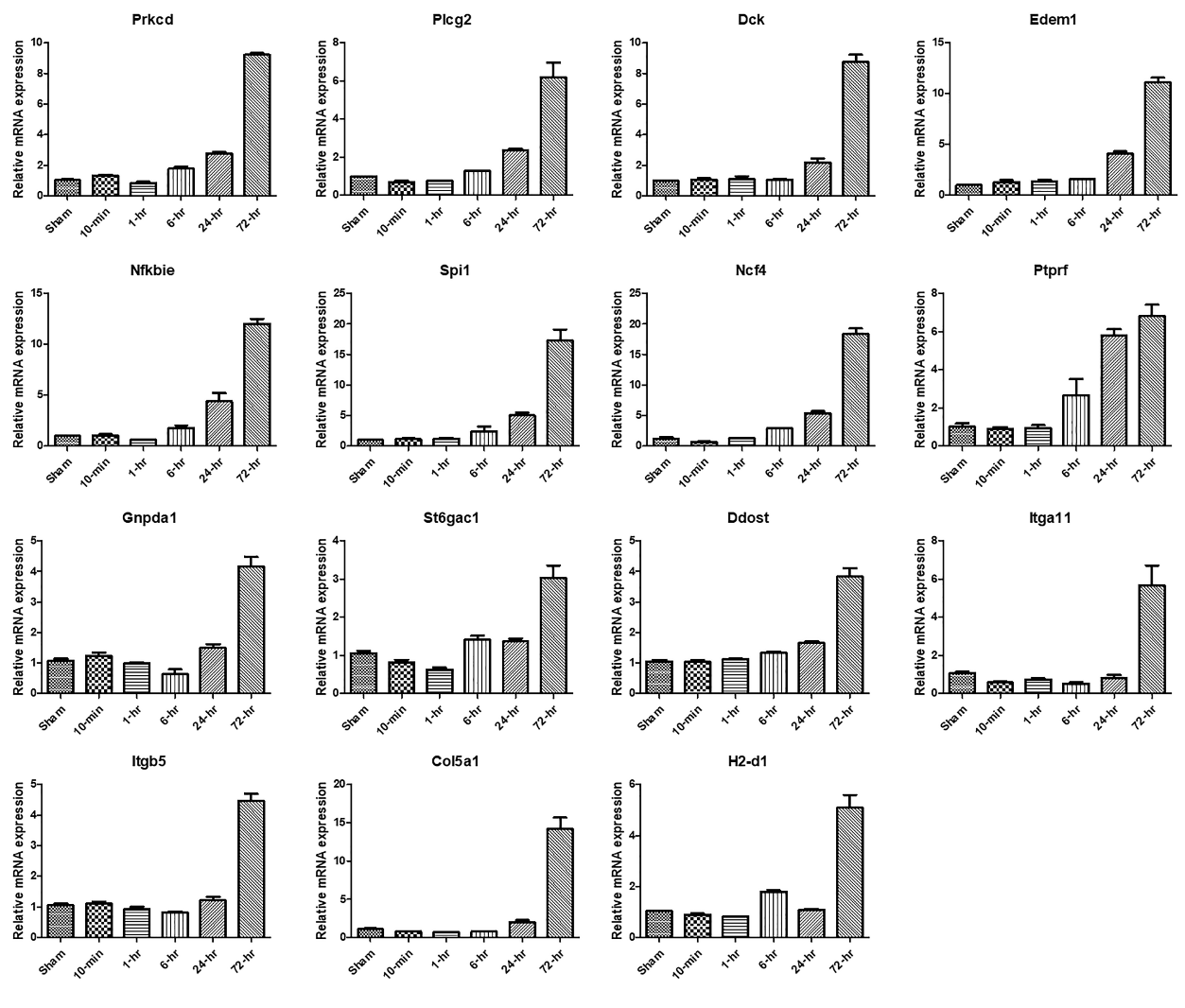


**Sup. Figure S5.** Validation of the promoter methylation expression of candidate genes by bisulfite sequencing PCR.


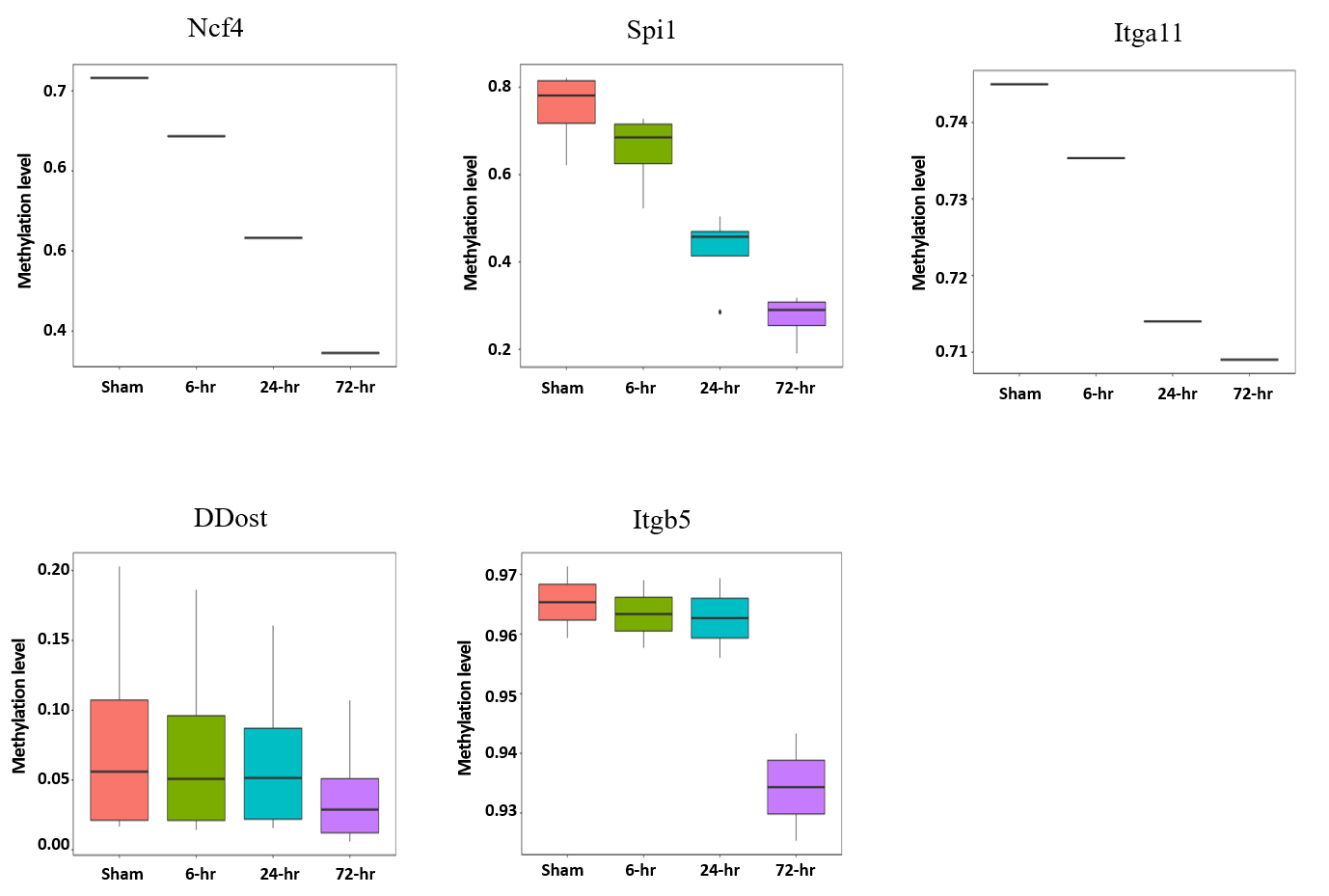


**Sup. Table S1** Candidate genes and involved KEGG pathways.

| Genes | KEGG_Pathways |
| --- | --- |
| Cyba | Osteoclast differentiation; Phagosome |
| St6gal1 | N-Glycan biosynthesis |
| Prkcd | Fc gamma R-mediated phagocytosis |
| H2-D1 | Cell adhesion molecules; Phagosome |
| Ddost | N-Glycan biosynthesis; Protein processing in endoplasmic reticulum; Metabolic pathways |
| Col6a1 | ECM-receptor interaction; PI3K-Akt signaling pathway; Focal adhesion |
| Gnpda1 | Amino sugar and nucleotide sugar metabolism; Metabolic pathways |
| Tec | Osteoclast differentiation |
| Plcg2 | Fc gamma R-mediated phagocytosis; Osteoclast differentiation; Leukocyte transendothelial migration; B cell receptor signaling pathway; Platelet activation; Metabolic pathways; NF-kappa B signaling pathway |
| Csf1r | Cytokine-cytokine receptor interaction; Hematopoietic cell lineage; PI3K-Akt signaling pathway; Osteoclast differentiation |
| Ncf4 | Phagosome; Osteoclast differentiation; Leukocyte transendothelial migration |
| Tubb6 | Phagosome |
| Dck | Pyrimidine metabolism; Purine metabolism; Metabolic pathways |
| Ptpn6 | B cell receptor signaling pathway; Adherens junction |
| Itgb3 | Osteoclast differentiation; ECM-receptor interaction; Focal adhesion; Regulation of actin cytoskeleton; Dilated cardiomyopathy; Platelet activation; Hypertrophic cardiomyopathy (HCM); Hematopoietic cell lineage; PI3K-Akt signaling pathway; Phagosome |
| Tcirg1 | Metabolic pathways; Phagosome; Lysosome |
| Itga11 | PI3K-Akt signaling pathway; ECM-receptor interaction; Focal adhesion; Regulation of actin cytoskeleton; Dilated cardiomyopathy; Hypertrophic cardiomyopathy (HCM) |
| Fcgr2b | Phagosome; Fc gamma R-mediated phagocytosis; Osteoclast differentiation; B cell receptor signaling pathway |
| Itgb5 | PI3K-Akt signaling pathway; Phagosome; ECM-receptor interaction; Focal adhesion; Regulation of actin cytoskeleton; Dilated cardiomyopathy; Hypertrophic cardiomyopathy (HCM) |
| Tgfb2 | Osteoclast differentiation; Dilated cardiomyopathy; Hypertrophic cardiomyopathy (HCM); Cell cycle; Cytokine-cytokine receptor interaction |
| Amdhd2 | Amino sugar and nucleotide sugar metabolism |
| Edem1 | Protein processing in endoplasmic reticulum |
| Nfkbie | B cell receptor signaling pathway |
| Map3k14 | NF-kappa B signaling pathway; Osteoclast differentiation; TNF signaling pathway |
| Col5a1 | Platelet activation; PI3K-Akt signaling pathway; Protein digestion and absorption; ECM-receptor interaction; Focal adhesion |
| Fos | TNF signaling pathway; Osteoclast differentiation; MAPK signaling pathway; Toll-like receptor signaling pathway; B cell receptor signaling pathway; T cell receptor signaling pathway |
| Thbs4 | PI3K-Akt signaling pathway; Phagosome; Focal adhesion; ECM-receptor interaction |
| Spi1 | Osteoclast differentiation |
| Ptprf | Adherens junction; Cell adhesion molecules (CAMs) |
| Tlr2 | PI3K-Akt signaling pathway; Phagosome; Toll-like receptor signaling pathway |
| Limk1 | Fc gamma R-mediated phagocytosis; Regulation of actin cytoskeleton |
| Fgf2 | Rap1 signaling pathway; Regulation of actin cytoskeleton; MAPK signaling pathway; PI3K-Akt signaling pathway |

**Sup. Table S2** Candidate genes selected to be verified the DNA methylation at promoters by BSP.

| **Excluded genes*** (n=11) |  | **Genes selected to be verified the DNA methylation at promoters by BSP** (n=21) |
| --- | --- | --- |
| Plcg2, Dck, Fcgr2b, Itgb3, Tgfb2, Thbs4, Tlr2, Fos, Amdhd2, Tec, Fgf2 |  | Gnpda1, St6gal1, Ddost, Csf1r, Itgb5, Col5a1, Col6a1, Itga11, H2-d1, Cyba, Tcirg1, Ptpn6, Ncf4, Map3k14, Edem1, Nfkbie, Prkcd, Ptprf, Spi1, Tubb6, Limk1 |

*Exclusion criteria: The changes of DNA methylation occurred after changes in gene expression
